# Assessment of the Mandatory Non-Financial Reporting of Romanian Companies in the Circular Economy Context

**DOI:** 10.3390/ijerph182412899

**Published:** 2021-12-07

**Authors:** Camelia-Daniela Hategan, Ruxandra-Ioana Pitorac, Nicoleta-Daniela Milu

**Affiliations:** 1 Department of Accounting and Audit, ECREB—East European Center for Research in Economics and Business, Faculty of Economics and Business Administration, West University of Timisoara, 16 Pestalozzi Street, 300115 Timisoara, Romania; camelia.hategan@e-uvt.ro; 2 Department of Economics and Economic Modeling, ECREB—East European Center for Research in Economics and Business, Faculty of Economics and Business Administration, West University of Timisoara, 16 Pestalozzi Street, 300115 Timisoara, Romania; ruxandra.pitorac@e-uvt.ro; 3 Doctoral School of Economics and Business Administration, Faculty of Economics and Business Administration, West University of Timisoara, 16 Pestalozzi Street, 300115 Timisoara, Romania

**Keywords:** reporting, non-financial information, corporate social responsibility, mandatory, circular economy

## Abstract

Between the circular economy and corporate social responsibility, there is an ever-closer connection. Non-financial reporting of social responsibility actions is based on the circular economy concept, so reporting contributes to increasing the level of disclosure of circular strategies. In this context, large companies are required to report non-financial information to understand their activities better. The paper’s objective is to assess the mandatory non-financial reporting of Romanian companies active in the non-financial sector for 2017–2019. The empirical analysis consisted of creating and awarding an evaluation score to the reports of the companies. An econometric model was tested using a feasible generalized least squares (FGLS) regression to identify the link of the obtained Score with a series of variables representing the characteristics of the companies: Information on a website (I), Foreign ownership (F), Private ownership (P), Listed company (L), Return on assets (ROA), and Return on equity (ROE). Research results highlight a positive correlation between Score and all variables statistically significant in the model. Our study empirically validated the link between non-financial reporting and financial performance. The practical implications for managers can be to focus on improving the quality of non-financial reporting by better presenting the sustainability actions in a circular economy context.

## 1. Introduction

Lately, companies worldwide have begun making increasing efforts to sustain the economic chain concerning sustainability regulations. As natural resources are increasingly scarce, air quality is deteriorating, water and soil are increasingly polluted, and international companies are more concerned with waste management using the best technologies. Bautista-Lazo and Short [1] consider that it is necessary for entities “to accept an economic model in which materials and energy from waste products are reintroduced into the economic system”. The concepts of “circular economy” and “sustainability” describe the extended framework for sustainable development, ensuring the company achieves healthy growth, both for itself and for society, by addressing environmental issues, degradation and lack of resources [2].

A successful company meets the needs of all stakeholders, both from a financial perspective (profit, turnover and capital) and a non-financial perspective (environmental protection, employee welfare and relationship with society). The company’s strategy to build its business based on the circular economy principles is, at the same time, an act of social responsibility. Thus, there is a direct link between the circular economy (EC) and corporate social responsibility (CSR), so that the way of applying the principles of the circular economy is reflected in the sustainability reports of companies [3].

In the context of evaluating a company’s performance, the challenge is for the company to report non-financial information (NFI). Following the transposition of European Directive 95/2014 (Directive) [4], the entities with an average number of more than 500 employees during the financial year must draw up a non-financial statement in the European Union. The non-financial information consists of issues regarding environmental protection, reducing social inequalities and employee protection, respect for human rights, the fight against corruption and bribery.

We decided to focus our research on Romanian companies because responsible business practices have increasingly accentuated in the last years [5]. The beginning of these practices has been given by multinational companies with their headquarters positioned in economically and socially well-developed areas, transferring organizational culture and practices. In Romania, companies have developed whose business objective is responsible, having activities that benefit to the environment and communities. The Romanian companies have developed the renewable energy industry, waste management and ecological products. Companies are aware that the model of the linear economy of production and global consumption is about to be overcome and becomes evident a sustainable change to a circular economy.

Non-financial reporting (NFR) transparency and credibility regarding the sustainable activities of Romanian companies have been a significant part of the analysis topics in the recent Romanian literature [5,6,7,8]. In this context, the paper’s objective is to assess the mandatory non-financial reporting of Romanian companies for 2017–2019. To fill the identified gaps in the literature regarding the measurement of disclosure’ degree of the Romanian companies, we develop a variable score by a specific criterion. Starting from analyzing companies in the non-financial sector that had to publish their reports, we develop an econometric model to identify the factors that may influence the quality and transparency of reporting, considering financial and non-financial variables.

The research contains an analysis of the available data on the level of compliance of Romanian companies with the Directive requirements from the perspective of transparency of information reported by companies. The paper can be a bibliographic source for accounting and sustainable development researchers for company management representatives to understand the need, and importance of reporting non-financial information on environmental, social and governance issues. Our study contributes to the existing research by synthesizing information on CSR reporting because the analysis was performed on an extensive sample of Romanian companies. The literature shows that previous studies have focused more on listed companies. Second, based on the available data, the variables included in the econometric model were selected to identify the characteristics of the analyzed companies.

The paper is organized as follows. In the next section, we present the literature review. In Section 2, the research methodology is described. Section 3 presents the results obtained together with the discussions generated by the research carried out. The final section contains the main conclusions, the limits of the research together with future research directions on this topic.

## 2. Literature Review

### 2.1. Circular Economy and Corporate Social Responsibility

The circular economy (CE) concept has become the European Union’s main priority as a trend of sustainable development, based on the relationship between the environment and the use of resources and between the economy and the well-being of society [9]. In the circular economy, the value of goods is kept as long as possible, waste and resources are kept to a minimum, and resources are capitalized in the economy until the product reaches the end of its useful life and can be used repeatedly to create additional value [10]. Scarpellini [11] shows that the circular economy focuses on economic improvement and environmental protection, social issues being less addressed, being justified by the fact that the EC offers substantial environmental and economic opportunities that involve social benefits. Assessing the social impact in an entity requires much effort and involves involving stakeholders and collecting various information [12]. In a recent study, Massaro et al. [13] show that the circular economy impacts the companies’ development, which involves identifying new sustainable solutions that positively impact the environment.

The concept of corporate social responsibility (CSR) is the basis for the development of social entrepreneurship (SE), according to the authors Urmanaviciene and Arachchi [14], who present in their paper that CSR and SE have as common goals the creation of social value. However, there are differences in maximizing corporate profit: corporate social responsibility pursues environmental, social and innovative actions pursuing the profitability of entities; SE pursuing the creation of social value; and managers being less interested in profit.

An increasingly close link has been created between the circular economy and corporate social responsibility. Non-financial reporting is based on the circular economy concept; thus, CSR reporting contributes to an adequate level of disclosure of companies’ circular strategies. Fortunati et al. [15] analyzed eight CSR reports from the cosmetics industry and found links between the circular economy and the CSR concept, even if the EC is not explicitly specified in these reports. The concept of social responsibility with the circular economy was connected through different terms: recycling, waste reduction, reuse, gas emissions, water and energy consumption [16].

According to Scarpellini et al. [17], there is a positive relationship between CE and CSR. The circular economy involves transforming a linear economic model into a circular model to reduce dependence on raw materials and energy and minimize the impact of companies’ activities on the environment. Better resources management will allow companies to enhance their social and environmental reporting and improve their relationship with consumers and stakeholders.

Skare and Golja [18] considered that the requirement for a company to develop economically is to take care of the environment through anti-pollution actions to respect society, which is the consumer of goods or services of the company, and which thanks to it the company carries out its activity. Janik et al. [19] analyzed 61 reports published by EU companies prepared according to Global Reporting Initiative (GRI) standards for 2018–2020. The study results showed that greenhouse gases (GHG) emissions and circular economy issues were briefly described. Thus, based on the indicators included in the reports, the companies did not sufficiently describe the methods they used to collect and analyze information on the effectiveness of the actions taken related to GHG and CE.

Oncioiu et al. [20] showed that, in Romania, the circular economy is seen as a strategy for the company, the circular economy activities of SMEs being at the beginning. The study results showed that after 2010 more than half of the analyzed companies were engaged in activities specific to the circular economy. The possibilities for the development of the circular economy can be achieved by creating a fiscal, legal or organizational framework, coupled with government actions to promote the circular economy. Thus, the promotion of ethical behavior belongs to each company philosophy [21].

### 2.2. CSR and Non-Financial Reporting

Previous research has examined whether economic, social and governance (ESG) factors affect a company’s economic and financial performance, with most studies showing a direct relationship that contributes to the value creation of companies and their resilience to economic shocks [22,23].

Both regulatory bodies and professional bodies are involved in establish quality non-financial reporting frameworks.

The European Commission pays more attention to potential developments in CSR, this aspect generating new regulations in the field. After the Directive application, certain aspects of reporting need to be improved, so following the consultations, the European Commission proposed revising the Directive in 2021 [24]. In the context of the green deal, the European commission regulated the taxonomy for sustainable activities. Thus, the regulation contains six environmental objectives, including the transition to the circular economy. Article 8 of the Taxonomy Regulation requires undertakings covered by the Directive to provide information on how and to what extent their activities are associated with economic activities that qualify as environmentally sustainable under technical examination criteria [25].

Accountancy Europe, the central body representing the accounting profession in Europe, considers that SMEs are much more exposed to risks if they do not introduce sustainability actions in their activity, considering the expectations of investors and customers [26]. For a company, it may be more expensive over time not to get involved in CSR actions because the requirements and expectations of stakeholders will be higher. Another body involved is the IFRS Foundation, which, following consultations on sustainability reporting, has committed itself to informally engaging in sustainability reporting to meet the need to inform stakeholders about improving coherence and comparability in CSR reporting. Thus, creating an appropriate set of standards would help companies publish information that would lead to greater transparency of sustainability initiatives to build public confidence [27].

Fiandrino and Tonelli [28] conducted a text analysis on the revision of the Directive and highlighted four main topics underlying the debate of the proposed legislative changes, namely the quality of NFI, standardization, materiality and assurance. In their study, La Torre et al. [29] examined the concept of accountability in the Directive context to provide a new critique and perspective for future research in NFR and advance future practice and policy. Therefore, the mentioned authors consider that it is necessary to rebuild trust by extending the mandatory practice of NFR beyond the traditional boundaries of accountability systems.

Most research on CSR disclosure has been conducted using data published by large companies or public interest entities and most research has analyzed the data of companies listed on the largest stock exchanges in Europe.

Raucci and Tarquinio [30] examined the effects produced on the disclosure of sustainability performance indicators by 31 Italian companies for 2017 after introducing the mandatory disclosure of NFI. Compared to the period before to the Directive’s application, the companies focused only on the indicators mentioned in the Directive without presenting other helpful information. Gazzola et al. [31] studied 63 Italian public interest companies that reported non-financial information between 2018 and 2020. The research objective was to assess the company’s level of sustainability derived from the company’s website and follow the presentation of United Nations sustainable development goals (SDGs) in published reports. The results showed a high capacity for companies’ sustainability increasing from one year to another derived from implementing active policies, although regional differences were found due to cultural differences. Venturelli et al. [32] analyzed the Directive’s impact on the comparability of non-financial statements on a sample of 70 Italian PIEs for 2016–2018. The results showed that the comparability of non-financial reports is too low. In another research, Caputo et al. [33] analyzed the non-financial reports of a sample of 145 Italian firms for the year 2017 to assess the transparency of environmental information. They found that the level of disclosure was influenced by corporate governance and the characteristics of the report. The intention to avoid disclosing unfavorable environmental information was also confirmed. Therefore, the revision of the Directive should also include these aspects.

Mion and Loza Adaui [34] performed a qualitative analysis of the reports of the top listed companies on the stock exchanges in Italy and Germany. The research objective was to highlight the changes resulting from the application of the Directive since 2017, comparing the reports published in 2016 and 2017. The results showed that the quality of sustainability reporting increased after the mandatory implementation of legislation and decreased the differences between the companies in the two countries. The main factors identified were the size of the company and the type of industry. Other research has shown that companies’ Directive implementation was done differently with these substantial differences between countries [35] and industries [36].

On the other hand, research has been carried out in the case of companies that are not of public interest. Krasodomska and Godawska [37] studied the behavior of a sample of non-public entities from Poland by analyzing the content of these companies’ websites. The results showed that CSR practices were briefly presented on websites, and there were mostly governance issues and environmental protection actions.

### 2.3. Non-Financial Reporting in Romania

The literature shows a gap in non-financial reporting by companies in the western and eastern EU, even though reporting practices have improved across countries, with significant influence being given by multinational companies [38].

Reporting on social responsibility activities should address the impacts at the organization and community level, as well as the results of the associated social impacts for assessing the financial performance of an organization [20]. Popescu and Popescu [39] found that Romanian entities are aware of the importance and advantages of corporate social responsibility correlated with increasing profit, performance and productivity.

The reporting of non-financial information needs to be harmonized and interconnected with financial information to provide credible, coherent and transparent information available to stakeholders [40,41,42,43]. A series of variables can be considered to analyze the correlations between the financial and non-financial indicators. Thus, quantification is more difficult in the case of non-financial indicators. Thus, the specialized literature was identified several ways of evaluating them. The most common way to evaluate was to create an index of non-financial information by giving a minimum and maximum score on different scales.

The non-financial reports published by the Romanian companies were analyzed in several studies. Marinescu [44] established the degree of compliance of the sustainability reports with the GRI standards of the Romanian companies available on the GRI Reporting website. The reports were evaluated by scoring them with scores from 1 to 3 in 2016–2018. The results show that the companies’ reports improve from one year to another, demonstrating a high level of adoption of sustainability practices according to the GRI reporting framework. Farcas [45] made a content analysis regarding the non-financial information to determine the degree of implementation of the Directive presented by a sample of 10 companies listed on BVB with Romanian majority capital in the period 2016–2019. The non-financial information was taken from the annual reports or other separate reports regarding the CSR information published on the BVB website, being awarded a score depending on the existence and transparency of this information (0: does not exist, 0.5: partially exists, 1: exists).

In most cases, non-financial information was extensive and provided a complete picture of the companies. Belenesi et al. [5] analyzed the reports’ content on non-financial information of companies listed on the Bucharest Stock Exchange (BVB) in 2017–2019. The degree of disclosure of the ESEG (environmental, social, economic and governance) indicator was also calculated. For the four indicators, 12 variables were used, granting scores depending on the disclosure of information (1: non-existent information, 2: poorly presented information, 3: information presented without details, 4: detailed information). The study results show that companies had slow but steady progress in non-financial information reporting. The publication of the social responsibility actions of the Romanian companies leads to the increase of the credibility of the interested parties.

## 3. Materials and Methods

### 3.1. Sample

The study is based on companies that in the period 2017–2019 had over 500 employees. This time frame was chosen because 2017 is the first year in which public interest companies that, at the balance sheet date, have over 500 employees required to report a non-financial statement, according to Romanian legislation. Starting with 2019, according to the new legislative changes, non-financial reporting becomes mandatory for all companies that have a minimum number of 500 employees.

The data of the companies were collected from the listefirme.ro website; this is a private database where official data are compiled. This database was used in previous research regarding financial indicators of Romanian companies [46,47]. The data were confronted on the official website of the Romanian Ministry of Public Finance to verify and complete the reliability of the data.

The first data collection stage identified companies in the non-financial sector with over 500 employees, excluding from the total number of companies the financial institutions due to their specific field of activity. Table 1 shows the dynamics of the companies for each year, the inputs representing the companies that in that year met the criterion of at least 500 employees, and the outputs are the companies that have less than 500 employees compared to the previous year.

**Figure 1:**
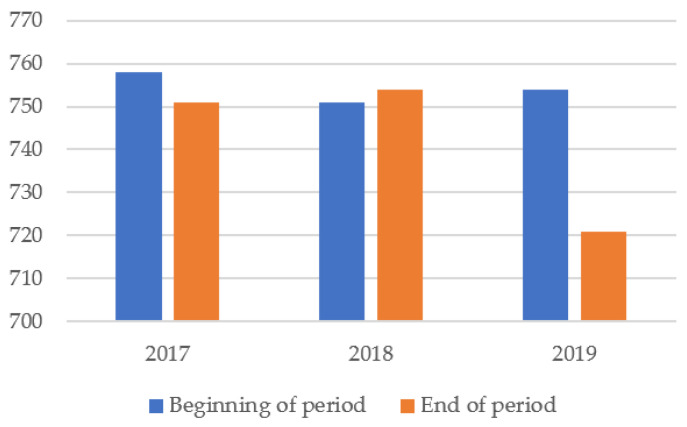
The evolution of the number of companies.

Table 1 shows a relative fluctuation of companies’ number each year, of ~10%, which shows a dynamic of company activity and the labor market in the context in which certain companies are below or above the limit of 500 employees. The most significant decline took place in 2019. Thus, in 2017, 751 companies were identified; in 2018, there were 754 companies, and in 2019, 721 companies were obliged to publish non-financial information (Figure 1).

The next step was to identify the companies that have maintained their number of employees over 500 in the three years to achieve the econometric model on panel data. The filtering process resulted in 603 companies, of which those with negative equity were excluded because they distort the financial indicators, reaching a final number of 542 companies included in the sample.

**Figure 2:**
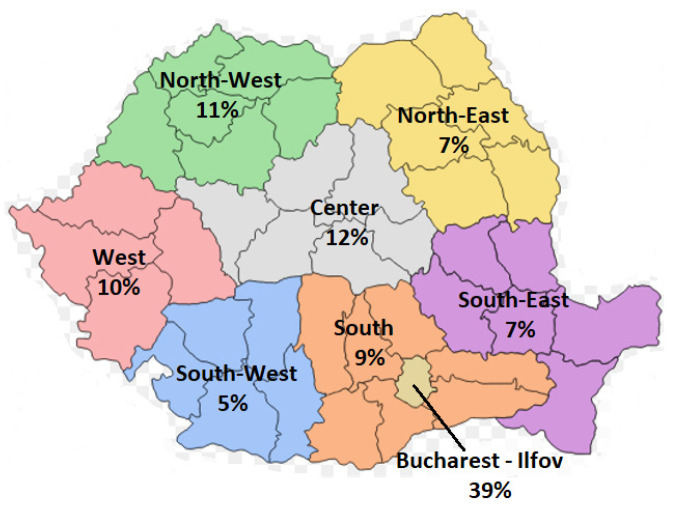
Distribution of companies by development regions.

To have an overview, the companies were grouped by development regions and field of activity. In Figure 2 are presented the distribution of companies by development regions.

From the results of Figure 2, 39% of the companies included in the sample were based in the Bucharest-Ilfov region, the most significant industrial area in Romania where all branches of industry are present. In the Center region, there are 12% of the companies, most of them of the manufacturing industry. On the third place is the North-West region with 11% of companies, the region is known for its natural richness and with a potential that is easy to exploit from a tourist and economic point of view. At the opposite pole is the South-West region, where only 5% of companies are located, followed by the South-East and North-East regions with 7%. For the South-East region, the Black Sea attracts investors, offering development through maritime transport. In recent years, the North-East region has also become preferred by investors due to cheaper labor.

**Figure 3:**
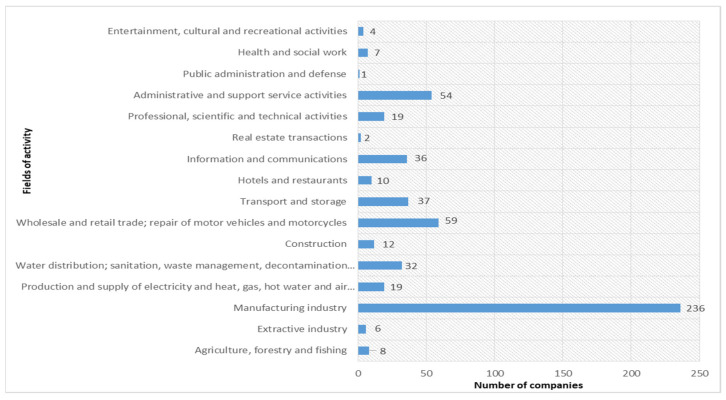
Activities.

The grouping of companies by fields of activity is presented in Figure 3.

According to Figure 3, it appears that most companies are in the manufacturing industry (236), the ranking is followed by companies in the field of trade (59) and those in the field of administrative services and support activities (54), such as human resources recruitment companies and security companies.

### 3.2. Methodology

To achieve the research objective of assessing the transparency degree of mandatory non-financial reports of Romanian companies, qualitative and quantitative research was conducted. An evaluation score of transparency degree by specific criteria was given for the qualitative analysis of the studied sample. The quantitative research consists of the analysis of the connection between the score given to the companies and the variables that represent the specific characteristics of the companies, which will be included in the proposed econometric model using an ordinary least squares (OLS) regression and a feasible generalized least squares (FGLS) regression. These types of regression were used in previous research on relationship between non-financial and financial performance [5,48,49]. In the absence of primary data for the authorized evaluation of non-financial reporting based on an independently calculated index, we developed a score based on our methodology.

**Figure 4:**
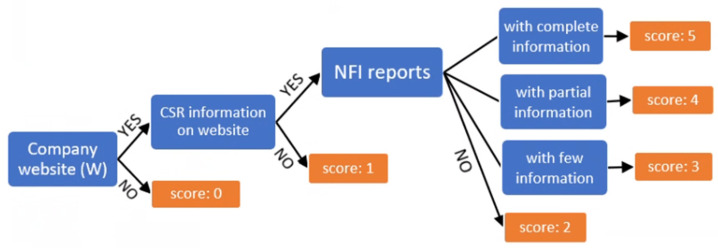
The stages and evaluation method the non-financial reporting.

In the second part of the sample data collection stage, non-financial information on companies’ corporate social responsibility was identified on their website to analyze the degree of implementation of the Directive’s requirements. The existence of a website and the online presentation of the related non-financial information in reports or sections precisely were verified for each company. After analyzing the existence of the information and how it is disclosed to the interested parties, each company was awarded an evaluation score (Figure 4).

The transparency of NFI reports was assessed using scoring methodology [50,51,52] for each specific criterion. Establishing the evaluation scale was based on previous research; Habek [53] and Matuszak and Różańska [54] used a 5-point scale (from 0 to 4) to analyze the quality of the reports. The non-financial information identified in the companies’ reports were grouped on the following social responsibility activities: employees, environment, relation with the community and innovation, so the resulting score is based on these elements. According to specific criteria met, each company was evaluated with a score of 1 to 5 based on a presentation of CSR actions on the website.

The structure and explanation of the qualitative evaluation of the information and non-financial reports are presented below in Table 2.

The most financial indicators used in the analysis are ROE and ROA [33,55]. Therefore, in the correlations with the non-financial indicators, previous research used ROE [56,57] and ROA [52,56,57]. Based on the literature review and the availability of data, the empirical research consists of a qualitative and quantitative analysis of the following indicators: Score (S), as the dependent variable, Information on a website (I), Foreign ownership (F), Private ownership (P), Listed company (L), Return on assets (ROA), Return on equity (ROE), as independent variables. The variables Employees (E) and Website (W) were included as control variables. For the quantitative analysis, we employed a panel data econometric model to study the influence of the independent variables on the dependent one.

The model tested had the following form:

S_i_ = α_i_ + β_1_I_i_ + β_2_F_i_ + β_3_P_i_ + β_4_L_i_ + β_5_ROE_i_ + β_6_ROA_i_ + β_7_E_i_ + β_8_W_i_ + ε_i_ (1)

To validate the econometric model, we performed an ordinary least squares (OLS) regression, an ANOVA test, the multicollinearity was tested using variance inflation factor (VIF), and we also tested for heteroskedasticity using Breusch–Pagan/Cook–Weisberg test. For the robustness check, we used the Hausman test, Pesaran’s test to check for cross-sectional dependence and to correct the heteroskedasticity and the autocorrelation of the residuals we used a feasible generalized least squares (FGLS) regression (with and without the control variables). All the tests and estimations were done in Stata Statistical Software: Release 17.

The description of the indicators is shown in Table 3.

## 4. Results

The quantitative analysis began with the descriptive statistics of the studied variables, as shown in Table 4.

The average score for the studied companies was 2.38. Half of the companies have CSR activities on their website, while 91% have a website. Regarding the ownership of the companies, 50% of the companies had foreign ownership and 85% of them had private ownership. Only 4% were listed on the stock exchange. The financial situation expressed by ROE showed that the average ROE was negative (−0.18), which means that the loss of companies significantly diminished their equity, the negative influence being mainly due to some state-owned companies. Thus, the average ROA was relatively low but still positive, 0.07, because the value of assets was much higher than the equity.

The correlation matrix between all the analyzed indicators, presented in Table 5, shows a strong positive correlation between the dependent variable and the information on the company’s’ website. Furthermore, there is a significant positive correlation with foreign ownership, with the fact that the company is listed, number of employees and the existence of a website and a significant negative correlation with private ownership.

For the econometric analysis, we performed an OLS regression to test for multicollinearity and obtained a mean VIF of 1.18, smaller than the threshold, which means there were no multicollinearity issues. ANOVA test revealed that F was 1045 at 1% level of significance, higher than the critical level; therefore, the model was valid. The Breusch–Pagan/Cook–Weisberg test results for heteroskedasticity revealed a chi2(1) of 157.74 with a *p*-value of 0.000, thus rejecting the null hypothesis. R-squared was 0.84, which means that there was a significant link between the variables. The modification of the independent variables can influence in a proportion of 84% the dependent variable, Score. The same methodology was used on the data from Manufacturing industry, and we obtained that the model is statistically valid (F was 1530.5 at 1% level of significance). The independent variables can influence the dependent one, in a proportion of 86%. Similar results were obtained from the models without the control variables (Employees and Website) for all companies and for the Manufacturing industry.

The results of the multivariate regressions, the coefficients of correlation together with t values from the Student’s test (in parentheses) and the significance level can be seen in Table 6. From all the independent variables, ROA was not statistically significant for all the analyzed companies. Furthermore, all the other independent variables were directly or indirectly correlated with the dependent one.

The regression results show that two indicators had a high impact on the dependent variable: the existence of information regarding CSR activities on the company’s website and if a company is listed or not. Overall, if there are information on the website, it could increase the score by 1.64 points, same as if the companies were listed, it can lead to an increase in score by 1.64. Furthermore, if the ownership is foreign, it can increase the score by 0.56, but if the ownership is private, it could decrease the score by 0.25. These results are consistent with previous studies [53,54].

The control variable, Employees, was statistically significant, but its influence was lower than the independent variables. The only control variable that had a higher coefficient was Website. If the companies have a website, it increases their score by 0.94 points.

As and additional test for the robustness of the results, we performed the Hausman test to check the fitness of the model and obtained a chi2(6) of 10.05 with a *p*-value of 0.1226, thus accepting the null hypothesis the preferred model is random effects. Afterwards, the cross-sectional dependence was tested using Pesaran’s test. We obtained a coefficient of −0.828 with *p*-value of 0.4079, and we accepted the null hypothesis that the residuals are not correlated, and each panel was strongly balanced. Therefore, we used a FGLS regression with homoscedastic panels, generalized least squares coefficients and no autocorrelation of residuals. Wald chi2(10) was 1646.4, higher than the threshold at a 1% level of confidence; therefore, the model was statistically significant. For a robustness check, we also performed the regression without the control variables.

We employed the same methodology on the companies from Manufacturing industry. For the Hausman test we rejected the null hypothesis, and the preferred model is with fixed effects (chi2(5) = 11.06 with *p* = 0.0019) and Pesaran’s test showed that the residuals were correlated (1.634 with *p*-value of 0.102). Therefore, we used a FGLS regression with homoscedastic panels, generalized least squares coefficients and adjusted Durbin Watson autocorrelation of residuals.

The results obtained with the FGLS model (Table 7) confirmed the results obtained with the previous model. The highest impact on the dependent variables had Information, followed by Listed companies. If the companies published the non-financial information on their website, the score might increase by 1.7, and if the companies were listed, it could increase the score by 1.5. The variable Private ownership had a negative influence on Score, if the companies had public ownership the score might increase by 0.78. The variable Foreign ownership had a positive impact on the dependent variable: if the companies had foreign ownership, the score might increase by 0.62. Romanian companies owned by private do not have a highly developed governance system, except for multinationals. Therefore, the relationship between the private indicator is negative because state-owned companies have a better governance system. The fact that the membership of companies in multinationals influences the relationship between the nationality of shareholders and the score, they use non-financial reporting as a marketing tool. These results are in accordance with the recent literature [53,54].

ROE was statistically significant at 10% level, but the coefficient was very small, 0.005, which means that ROE has the lowest contribution to the quality of sustainability reporting. Similar results were obtained without the control variables. For ROA, the influence on Score is negative, in accordance with some of the previous studies, but the result is insignificant [52,57].

For the companies from the manufacturing industry the highest influence on the dependent variable had Information (if the companies published the information on website the score might increase by almost 2 points) followed by Listed (if the companies were listed the score could increase by 1.7). The difference for this model was the variable Private ownership, which had a positive impact, so if the companies had private ownership the score increased by 0.5. ROA became statistically significant in line with previous research [33], if ROA increased by 1%, the score might increase by 1.1 points.

## 5. Discussion

Romania is a member of the European Union; therefore, it is required to apply the NFR Directive. Our study is essential to reflect the stage of NFI reporting of Romanian companies to find the level of disclosure of reports compared with other countries EU members. Transitioning to the circular economy is a priority of the European Commission; consequently, the companies and the communities need to apply this fact by creating a permanent dialogue [58].

From the qualitative analysis of the reports to evaluate them, gaps, inconsistencies or overlaps of information were found, so it was necessary to synchronize the financial information with the non-financial ones [59]. The Romanian company with the best sustainability reporting practices is OMV Petrom Group, which has integrated reporting that provides a complete and balanced overview of the company’s position and performance, responsibility, conciseness, materiality and reliability [60]. OMV Petrom was recognized in 2020 as the best Bucharest Exchange Trading (BET) index company in Romania for gender equality [61].

Based on the assessment of the mandatory NFR, it was found that the average score of 2.38 is a low value which shows that most companies have a reduced degree of reporting of non-financial information [62], even if there is slow progress from one year to another, this situation being consistent with the results obtained by Belenesi et al. [5]. The progress is due to transparency’ improvement of the reporting of the same companies and the fact that new companies with satisfactory reporting were included in the sample during the period.

The socially responsible companies from the sample included in their reports the way of assessing the sustainability risks by referring to the circular economy as an aspect of materiality. The implications of the circular economy are also found in the social field, contributing to changing behavior and improving the quality of life. It will provide economic benefits to society by reducing social inequalities and creating jobs.

The companies with foreign capital present in Romania had a higher score because most were multinationals that implemented the same reporting policies as in the country of origin. Private equity has a negative relationship with the evaluation score of non-financial reporting of companies, which means that state-owned companies with a share of 15% in the sample had a higher degree of transparency than private companies, even if many were multinationals.

It is known that listed companies must adopt governance codes that require them to disclose non-financial information. Thus, most research has been performed on the data presented by these companies because their availability is higher than non-listed, and the data can be processed more quickly. The results obtained are correlated with other research that identified that Romanian companies listed on the BVB comply with Directive rules on non-financial reporting, even if some report to a greater extent than others and they showed a practice of reporting issues related to employees, environment, risks and business model [46,62].

Due to the sample characteristics, the results did not confirm the initial expectations, respectively, that all two indicators (ROE and ROA) influence the level of disclosure of non-financial reporting. The correlation of the Score with the financial indicators is significant only in the case of ROE for all sample, in line with the results obtained by Batae [57]. For manufacturing industry, a positive relationship was found with ROA, in line with previous research [33,52,57].

The sample’s analyzed had a high degree of concentration regarding the location, and most companies were based in the Bucharest-Ilfov development region (39%). Considering the classification by industries, 44% of the companies had activities of the manufacturing industry. Previous research has shown that the quality of sustainability reports differs by industry [34,36,63]. We tested the model by industry, which was the most prevalent by companies in the manufacturing industry. The results obtained confirmed these differences, namely that in the case of companies in the manufacturing industry, slightly different results were obtained compared to the total sample, especially in the relationship between the score and the financial indicators. Therefore, the financial performance of companies influences to a certain extent the degree of transparency of non-financial information.

As mentioned earlier, there has been a gap in CSR reporting between Western and Eastern European countries. Romania is a country in Eastern Europe. In order to compare the situation of Romanian companies regarding CSR, companies that also come from this part of Europe should be studied because the effects of communism are still visible even after 30 years [64]. Poland is one of the countries with which several comparisons have been made. Thus, from their study on non-financial reporting prepared before applying the NFR Directive conducted on the case of Romanian and Polish non-financial companies Dumitru et al. [65] found that Romanian companies had a higher disclosure score than those in Poland. Our results confirm previous research, namely that listed companies have a higher degree of transparency than unlisted companies.

According to the theory of stakeholders, which underlies CSR actions, if a company does not pay special attention to all stakeholders, it can have severe difficulties remaining on the market in the long run. The link between EC and CSR has not been intensively researched so far, and studies are needed to argue the interconnections between the two concepts theoretically [3,15,66]. Through our study, we evaluated the non-financial reporting of companies to show their status and how companies understood to inform stakeholders. CSR activities are considered the “ground” of the circular economy [66], and the better they are communicated to stakeholders, moreover, companies will benefit in the future.

The transition from the linear to the circular economy involves additional costs for companies. However, moving to the EC will benefit both companies and stakeholders. Thus, managers who pay more attention to CSR practices are aware that they will improve the brand and strengthen customer trust. Thus, the practical implications of our study are that managers who have focused on improving the quality of non-financial reporting by better presenting sustainability actions can realize that companies are prepared for the transition to the circular economy [67]. Furthermore, companies that have not presented sustainability actions may be motivated to start them because they will be helpful in the process of transition to EC.

## 6. Conclusions

Previous research shows a high level of integration between corporate social responsibility and the circular economy that can benefit companies by reducing costs, access to capital, customer relations and innovation [66]. The EC builds on the sustainable concepts of CSR and then turns them into practices [68]. These CSR practices based on the circular economy concept are included in the non-financial reporting. Therefore, the reporting of CSR actions contributes to an appropriate level of disclosure of companies’ circular strategies.

The interest of companies on CSR actions are growing, and researchers [69] have identified 2011 as an important milestone in the maturity of sustainability reporting. Most research has investigated the degree of implementation of Directive by European companies and the quality of NFR and found that there are still many improvements to be made. Some authors believe that integrated reporting should be moved to reduce the ambiguity of the information reported [70].

This paper presented the characteristics of Romanian companies with more than 500 employees for 2017–2019. The data analysis showed significant differences between the companies in the case of all the analyzed indicators, which shows that sustainability is an essential factor in increasing the confidence for a sustainable business. The econometric model results showed a positive correlation between score and all variables statistically significant in the model. Our study empirically validated the link between non-financial reporting and financial performance, in the case of ROE for all companies included in sample. The positive correlations between the score and the two financial indicators ROE and ROA were validated for manufacturing industry. Other independent variables significantly influenced the score obtained by each company following the evaluation of the degree of transparency of the non-financial reports.

The application by the companies of the circular economy principles will suppose among others, the reporting of their specific activities, respectively of the information regarding the green acquisitions, environmental actions, and social involvement in the community. All these reported activities can contribute to creating a database and the establishment of evaluation criteria regarding the level of involvement of each company in the context of the new action plan for the circular economy adopted by the European Commission.

In the future, the credibility of non-financial reporting can be raised by the practices used in providing information [71], this topic being in full debate. We can conclude with the words of Carroll [72], who looked back at the CSR concept and mentioned that “it has had a robust past and will have an upbeat and optimistic future”.

The paper can be a bibliographic source for researchers in corporate social responsibility, non-financial reporting, and circular economy, both through the indicators used and the econometric model tested.

Our study presented some limitations that indicate the potential of future research. First, the results are limited to the chosen sample, namely, that only companies with mandatory non-financial reporting were included, but from the literature results, the number of companies that publish voluntary information was relatively small. The second limitation is that the companies’ governance indicators associated with financial performance were not included, the reason being that many companies did not publish this information on the website. A third limitation is methodological. Thus, when interpreting the results, it must be considered that the data were taken from secondary sources, and the evaluation of the transparency degree may have a subjectivity.

Future research directions can be oriented to extend the period with the new data and can be made comparative analyzes on several countries with the inclusion of other relevant variables. From a methodological point of view, the assessment method could be improved by detailing the score used on the transparency degree of non-financial information by including several factors that can be considered. Furthermore, it is possible to analyze how the companies adhered to SDGs and transitioned to the circular business model to broaden the research perspective.

## Figures and Tables

**Table 1:** The dynamics of the number of companies.

Year	Beginning of Period	Inputs	Outputs	End of Period
2017	758	74	81	751
2018	751	64	61	754
2019	754	53	86	721

**Table 2:** Description of the score.

Score	Description
1	lack of non-financial information in separate corporate social responsibility reports/statements or sections in the annual reports;
2	limited information, the information is mentioned on the website, but is not described in a section of a report or in a separate report;
3	some information is presented in the annual reports, but with deficiencies, the information is described in insufficient coverage;
4	almost comprehensive information, a good description of the information, but there is no connection with other sections of the report, this information can be found in separate reports or sections of the annual reports;
5	complete information, the information presented is comprehensive, with a clear link to other sections of the report.

**Table 3:** Description of indicators.

Indicator	Code	Description
Score	S	1 to 5
Information on a website	I	1—Yes; 0—No
Foreign ownership	F	1—Yes; 0—No
Private ownership	P	1—Yes; 0—No
Listed company	L	1—Yes; 0—No
Return on assets	ROA	Own computation based on financial statements
Return on equity	ROE	Own computation based on financial statements
Employees	E	Number of employees
Website	W	1—Yes; 0—No

Source: Own Processing.

**Table 4:** Descriptive statistics.

Variables	Obs.	Mean	Std. Dev.	Min	Max
S	1626	2.3862	1.5399	1	5
I	1626	0.5043	0.5001	0	1
F	1626	0.5092	0.5000	0	1
P	1626	0.8597	0.3473	0	1
L	1626	0.0405	0.1974	0	1
ROE	1626	−0.1815	9.1171	−280.06	4.03
ROA	1626	0.0716	0.1111	−0.72	0.77
E	1626	1722.83	2392.755	500	23404
W	1626	0.9151	0.2787	0	1

Source: Own computation using Stata 17.

**Table 5:** Correlation matrix.

Obs.	S	I	F	P	L	ROE	ROA	E	W
S	1								
I	0.6411 ***	1							
F	0.3294 ***	0.3849 ***	1						
P	−0.0540 **	0.1310 ***	0.4114 ***	1					
L	0.2116 ***	0.0356	−0.1347 ***	−0.1054 ***	1				
ROE	0.0091	−0.0332	0.0037	0.0443 *	0.0058	1			
ROA	−0.0207	0.0143	0.0998 ***	0.1656 ***	−0.0367	0.0882 ***	1		
E	0.2206 ***	0.1524 ***	0.0927 ***	−0.1248 ***	0.0405	0.0054	−0.0644 ***	1	
W	0.2570 ***	0.3072 ***	0.0718 ***	−0.1039 ***	0.0626 **	−0.0128	−0.1123 ***	0.0570 **	1

*—10% level of significance, **—5% level of significance, ***—1% level of significance; Source: own computation using Stata 17.

**Table 6:** Results of OLS regression.

Obs.	R^2^	F	I	F	P	L	ROE	ROA	E	W
All companies										
1626	0.8402	1045 ***	1.6273	0.5606	−0.1259	1.6055	0.0041	0.3613	0.0008	1.1782
			(26.74) ***	(9.69) ***	(−1.64) *	(13.99) ***	(4.56) ***	(1.40)	(5.48) ***	(15.12) ***
1626	0.7979	1246 ***	2.1367	0.5380	0.8036	2.041	0.0027	0.3873	-	-
			(38.93) ***	(9.74) ***	(19.97) ***	(14.80) ***	(1.41)	(1.38)		
Manufacturing industry										
708	0.8668	1530.5 ***	1.9851	0.4810	0.5340	1.6687	0.0056	0.8792	0.0001	0.2386
			(23.11) ***	(5.80) ***	(4.44) ***	(10.39) ***	(11.52) ***	(2.05) **	(3.17) ***	(2.08) ***
708	0.8617	1926 ***	2.098	0.569	0.831	1.718	0.0059	0.784	-	-
			(24.07) ***	(7.36) ***	(13.86) ***	(10.26) ***	(12.14) ***	(1.80) *		

*—10% level of significance, **—5% level of significance, ***—1% level of significance; Source: own computation using Stata 17.

**Table 7:** Results of FGLS regression.

Wald CHI2 (10)	I	F	P	L	ROE	ROA	E	W
All companies								
1643.5 ***	1.7116	0.6293	−0.7830	1.517	0.0057	−0.0855	0.0005	0.1987
	(27.64) ***	(9.76) ***	(−8.89) ***	(10.94) ***	(1.93) *	(−0.34)	(4.68) ***	(1.92) *
1593.5 ***	1.7799	0.6648	−0.8731	1.5491	0.0061	−0.1912	-	-
	(30.10) ***	(10.30) ***	(−10.07) ***	(11.10) ***	(11.10) ***	(−0.77)		
Manufacturing Industry								
3433.7 ***	1.9958	0.4814	0.5123	1.663	0.011	1.167	0.001	0.225
	(18.93) ***	(4.44) ***	(3.31) ***	(7.83) ***	(1.81) *	(2.07) **	(4.19) ***	(1.47)
3250.7 ***	2.110	0.569	0.792	1.711	0.011	1.093	-	-
	(20.92) ***	(5.24) ***	(8.46) ***	(7.97) ***	(1.79) *	(1.91) **		

*—10% level of significance, **—5% level of significance, ***—1% level of significance; Source: own computation using Stata 17.

## Data Availability

The data presented in this study are available upon request from the corresponding author.
